# Clinicians’ adherence to guidelines for the preoperative management of direct oral anticoagulants in a tertiary hospital: a retrospective study

**DOI:** 10.1186/s12871-023-02276-w

**Published:** 2023-09-15

**Authors:** Jean Terrier, Amélie Mach, Pierre Fontana, Fanny Bonhomme, Alessandro Casini

**Affiliations:** 1 Division of General Internal Medicine, Geneva University Hospitals, Geneva, Switzerland; 2 Division of Clinical Pharmacology and Toxicology, Geneva University Hospitals, 4 Rue Gabrielle Perret-Gentil, 1205 Geneva, Switzerland; 3 Division of Angiology and Hemostasis, Geneva University Hospitals, Geneva, Switzerland; 4 Division of Anesthesiology, Geneva University Hospitals, Geneva, Switzerland

**Keywords:** DOAC, Preoperative, Periprocedural, Management, Anticoagulant, Surgery

## Abstract

**Introduction:**

Despite clear, relatively easy-to-use guidance, many clinicians find the preoperative management of direct oral anticoagulants (DOACs) challenging. Inappropriate management can delay procedures and lead to haemorrhagic or thromboembolic complications. We aimed to describe preoperative management practices regarding DOACs in a tertiary hospital and clinicians’ adherence to in-house recommendations.

**Method:**

We included all patients being treated with DOACs who underwent elective surgery in 2019 and 2020 (*n* = 337). In-house recommendations for perioperative management were largely comparable to the 2022 American College of Chest Physicians guidelines.

**Results:**

Typical patients were older adults with multiple comorbidities and high thrombotic risk stratification scores, and 65.6% (*n* = 221) had not undergone recommended preoperative anticoagulation management protocols. Patients operated on using local anaesthesia (adjusted OR = 0.30, 95%CI 0.14–0.66; *p* < 0.01) were less likely to have been treated following institutional recommendations, but no association between their procedure’s bleeding risk and adherence was found. Clinicians’ failures to adhere to recommendations mostly involved late or non-indicated interruptions of anticoagulation treatment (*n* = 89, 26.4%) or inappropriate heparin bridging (*n* = 54, 16.0%). Forty-five (13.3%) procedures had to be postponed. Incorrect preoperative anticoagulation management was directly responsible for 12/45 postponements (26.7% of postponements).

**Conclusion:**

This study highlights clinicians’ low adherence rates to institutional recommendations for patients treated with DOACs scheduled for elective surgery in a tertiary hospital centre. To the best of our knowledge, this is the first clinical study addressing the issue of clinicians’ adherence to guidelines for the preoperative management of DOACs. Going beyond the issue of whether clinicians are knowledgeable about guidelines or have them available, this study questions how generalisable guidelines are in a tertiary hospital managing many highly polymorbid patients.

Further studies should identify the causes of poor adherence.

**Supplementary Information:**

The online version contains supplementary material available at 10.1186/s12871-023-02276-w.

## Background

Direct oral anticoagulants (DOACs) have become the medication of choice for the treatment and prophylaxis of deep vein thrombosis (DVT) and pulmonary embolism (PE), as well as for reducing the risk of stroke and systemic embolism in non-valvular atrial fibrillation (AF) [1, 2]. However, patients treated with DOACs often face increased bleeding risks (due to advanced age, polymorbidity or polymedication), and their anticoagulant therapies are frequently interrupted for surgical or non-surgical procedures [3, 4]. Each year, one in six patients with AF—an estimated six million patients worldwide—require perioperative anticoagulant management [4]. Appropriate management of this interruption is critical to avoiding periprocedural bleeding or ischaemic events. Correctly timing interruptions is essential and can affect the organisation and planning of procedures for both physicians and patients. Several guidelines validated by the Perioperative Anticoagulation Use for Surgery Evaluation (PAUSE) study suggest managing the interruption of preoperative anticoagulation based on renal function and surgery bleeding risk (Supplementary table 1 in Additional file 1) [5–7]. The PAUSE protocol recommends that the interruption of anticoagulation therapy should occur one to two days before surgery for patients taking rivaroxaban and apixaban, depending on their risk of bleeding, and one to four days before surgery for patients taking dabigatran, depending on their renal function. The rates of thromboembolic and haemorrhagic complications reported in the PAUSE study were low, with major bleeding observed in less than 2% of patients and ischaemic stroke observed in less than 0.5%, thereby confirming this approach’s safety [7]. Furthermore, the approximately 94% adherence rate to the pre- and postoperative DOAC management protocol also supports this approach’s generalisability [7]. However, polymorbid patients undergoing polymedication and/or with renal insufficiency were underrepresented. Indeed, across the three DOAC cohorts, only 5–8% of patients were prescribed a P-glycoprotein or cytochrome P450 3A4 inhibitor or inducer and had a mean creatinine clearance above 87.7 ml/min. In addition, only a third of the patients included underwent a high-bleeding-risk procedure [7]. The present study aimed to describe the preoperative management practices for DOACs in a tertiary hospital, compare this management with standard institutional protocols based on the most recent guidelines and evaluate those practices’ effects on planned procedures.

## Methods

### Study design

We conducted a retrospective study based on a cohort of patients treated using DOACs who underwent elective surgery during 2019 and 2020. The data collection was approved by the cantonal research ethics commission (CCER 2021–00639). Study inclusion criteria were being ≥ 18 years old, undergoing anticoagulation therapy with any DOAC (i.e. rivaroxaban, apixaban, edoxaban or dabigatran) and being scheduled for elective surgery. Exclusion criteria were the absence of information on the date of surgery, flutter ablations, cardioversions and contraindications to surgery detected at the anaesthesia consultation.

We extracted data from patients’ electronic medical files, mostly during anaesthesia consultations, including patient characteristics (age, sex, body mass index, CHA_2_DS_2_ and/or CHA_2_DS_2_-VASc risk stratification scores for patients with AF), comorbidities (heart failure, hypertension, diabetes, stroke, transient ischaemic attack, coronary artery disease, lower-limb artery disease, a prosthetic valve, mitral regurgitation, thromboembolic venous disease, active cancer), laboratory values (haemoglobin, platelets, serum creatinine, creatinine clearance), type of DOAC and dosing, surgical bleeding risk as defined by the International Society on Thrombosis and Haemostasis (ISTH) Guidance Statement (Supplementary Table 2, Additional file 1) [8], type of anaesthesia (general, neuraxial, local or other), and intervention postponements and their causes.

Type of anticoagulation therapy, the risk of the planned surgery and the proposed anticoagulation interruption interval were collected to determine how closely clinicians had adhered to institutional recommendations (our main objective) [9]. These recommendations accorded with the most recent guidelines from the American College of Chest Physicians (ACCP) [5], except for its requirement to institute heparin bridging in the event of a recent thromboembolic event (< 3 months prior) (Supplementary Table 3, Additional file 1).

Furthermore, minimal and low-risk procedures were considered together, as the PAUSE protocol suggests [7], whereas the ACCP guidelines propose continuing DOACs during minimally risky procedures [5]. The secondary objective was to determine the prevalence of procedural postponements due to poor anticoagulation management.

### Statistics

We performed a descriptive statistical analysis of our data, with continuous data shown as means plus standard deviations and binary data shown as proportions. We measured associations between clinicians’ adherence to the ACCP guidelines and other relevant covariates using an unadjusted logistic regression. Less than one covariate for every ten events were used to keep the risk of overfitting low. Significant covariates obtained using univariate regression analysis (*p* < 0.2) were then tested using multivariate analysis and were finally included if they were significant after adjustment (*p* < 0.05). All these analyses were performed using Stata Statistical Software (Release 17, StataCorp LLC, College Station, TX, USA).

## Results

### Screening and inclusion

**Fig. 1:**
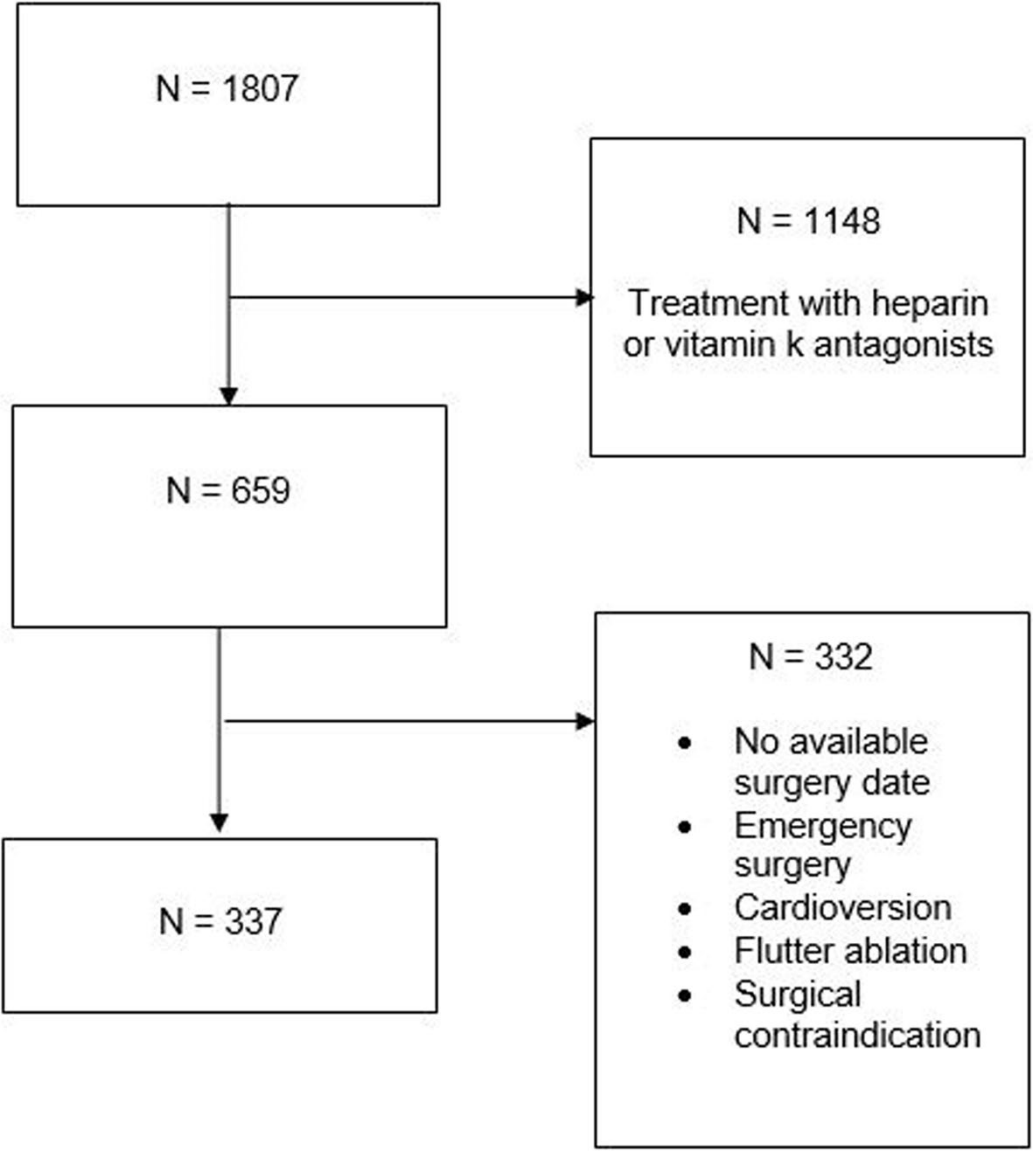
Inclusion flowchart

The study flowchart (Fig. 1) shows that a total of 1807 anticoagulated patients scheduled to undergo elective surgery were screened; 1148 did not meet the inclusion criteria due to anticoagulation therapy using vitamin K antagonists (VKA) or heparin (e.g. for venous thromboembolic disease in cancer patients). Of the remaining 659 patients, 322 were excluded after their anaesthesia consultation for various reasons (urgent surgery, undetermined operative date, cancelled procedure, lost to follow-up).

### Patient characteristics

The main characteristics of the patients included are presented in Table 1. Mean patient age was 74 years old (range: 19–97). There was a sex imbalance of approximately two males to one female. The population was predominantly overweight, with multiple risk factors and cardiovascular comorbidity. Patients with AF were characterised by CHA_2_DS_2_-VASc scores showing moderate risk stratification. Most patients were on DOACs due to AF (71.5%), with rivaroxaban being the DOAC most used (36.1%, *n* = 189), followed by apixaban (31.5%, *n* = 106), edoxaban (9.2%, *n* = 31) and dabigatran (3.6%, *n* = 12). A minority of patients (12.2%, *n* = 41) were also undergoing antiplatelet therapy. Creatinine clearance (CrCl) rates were only available for 211 (62.6%) patients, and of these, 51 (25%) had a CrCl < 50 ml/min (according to the Cockcroft–Gault equation).

**Table 1:** Study patients’ main characteristics

**Patient characteristics**	***n* = 337**
Mean age (mean ± SD) (y)	73.9 ± 11.8
Sex (n, %)	123 females (36.5%)
	214 males (63.5%)
BMI (kg/m^2^)	26.4 ± 4.8
**Risk stratification**	
CHA_**2**_DS_2_-VASc (mean ± SD)	3.8 ± 1.6
**Indication for DOAC**	
VTE (n, %)	96 (28.5)
AF (n, %)	241 (71.5)
**Antiplatelet therapy**	
Yes (n, %)	41 (12.2)
No (n, %)	296 (87.8)
**Type of DOAC**	
Rivaroxaban (n, %)	188 (55.8)
Apixaban (n, %)	105 (31.2)
Edoxaban (n, %)	31 (9.2)
Dabigatran (n, %)	13 (3.9)
**Comorbidities**	
Heart failure (n, %)	26 (7.7)
Hypertension (n, %)	227 (67.4)
Diabetes (n, %)	60 (17.8)
Stroke < 3 months (n, %)	5 (1.5)
Stroke > 3 months (n, %)	40 (11.9)
VTE < 3 months (n, %)	12 (3.6)
VTE > 3 months (n, %)	84 (24.9)
Coronary disease (n, %)	83 (24.6)
Bioprosthetic valve (n, %)	7 (2.1)
Cancer (n, %)	57 (16.9)
No comorbidities (n, %)	28 (8.3)
**Renal function (Cockcroft–Gault)**	
CrCl ≥ 50 ml/min (n, %)	160 (75.8)
CrCl = 30–49 ml/min (n, %)	43 (20.4)
CrCl < 30 ml/min (n, %)	8 (3.8)

*SD* standard deviation, *BMI* body mass index, *DOAC* direct oral anticoagulant, *VTE* venous thromboembolism, *AF* atrial fibrillation, *CrCl* creatinine clearance

### Surgical procedures and anaesthesia

Almost half of the patients underwent high-bleeding-risk surgery, whereas the other half was well-balanced between low-to-moderate and minimal-risk procedures (Table 2). Almost two-thirds of patients underwent general anaesthesia, and about a quarter underwent local anaesthesia (peripheral anaesthesia). Neuraxial and other anaesthetic methods (sedation only, hypnosis, etc.) were anecdotal.

**Table 2:** Bleeding risk for surgical procedures and type of anaesthesia

**Surgical procedures: bleeding risk**	***n* = 337**
High (n, %)	154 (45.7)
Low–Moderate (n, %)	96 (28.5)
Minimal (n, %)	87 (25.8)
**Type of Anaesthesia**	
General (n, %)	227 (67.4)
Local (n, %)	91 (27.0)
Neuraxial (n, %)	15 (4.5)
Other (n, %)	4 (1.2)

###  In-house recommendations adherence

**Fig. 2:**
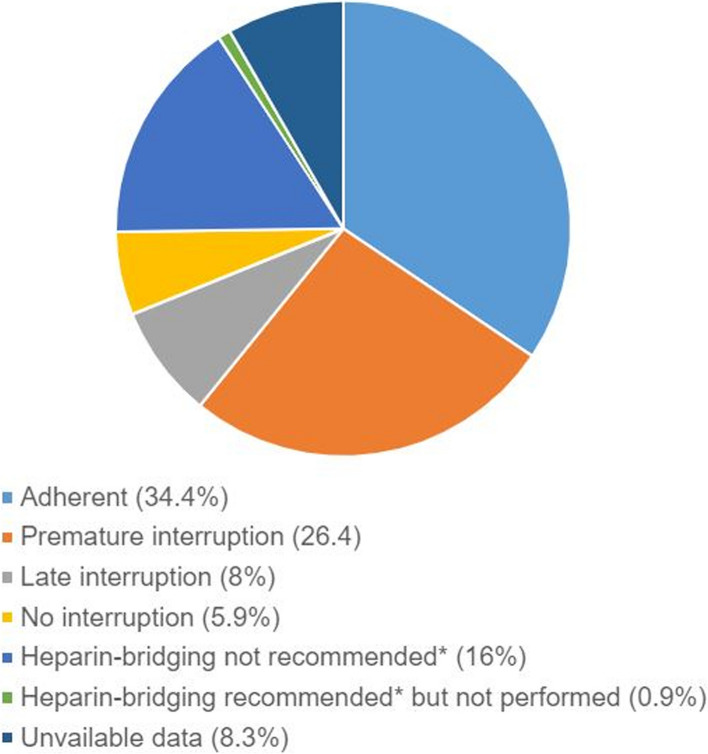
Rate of adherence to ACCP guidelines following institutional recommendations given to patients and causes of clinician’s non-adherence. *According to institutional recommendations [9]

Only 116 patients (34.4%) had followed our institution’s anticoagulation management recommendations, which are mainly based on the ACCP guidelines. Of the 221 (65.6%) who had not followed this protocol (Fig. 2), the majority (*n* = 89, 26.4%) had had their treatment prematurely interrupted (DOAC stopped before the recommended D -1 or D -2 before surgery), 54 (16.0%) underwent heparin bridging despite it not being recommended, 28 (8.3%) cases had no data (*n* = 28, 8.3%) and 27 (8.0%) patients had their treatment interrupted too late (on D -1 instead of D -2). Overall, 19/54 (35.2%) patients who underwent heparin bridging had had a thromboembolic event < 3 months previously, and 35/54 (64.8%) had had a thromboembolic event > 3 months previously. Twenty patients (5.9%) did not have their anticoagulant treatment interrupted, and only three (0.9%) patients had not undergone heparin bridging according to our institutional recommendations (thromboembolic event < 3 months previously).

### Determinants of adherence to guidelines

**Fig. 3:**
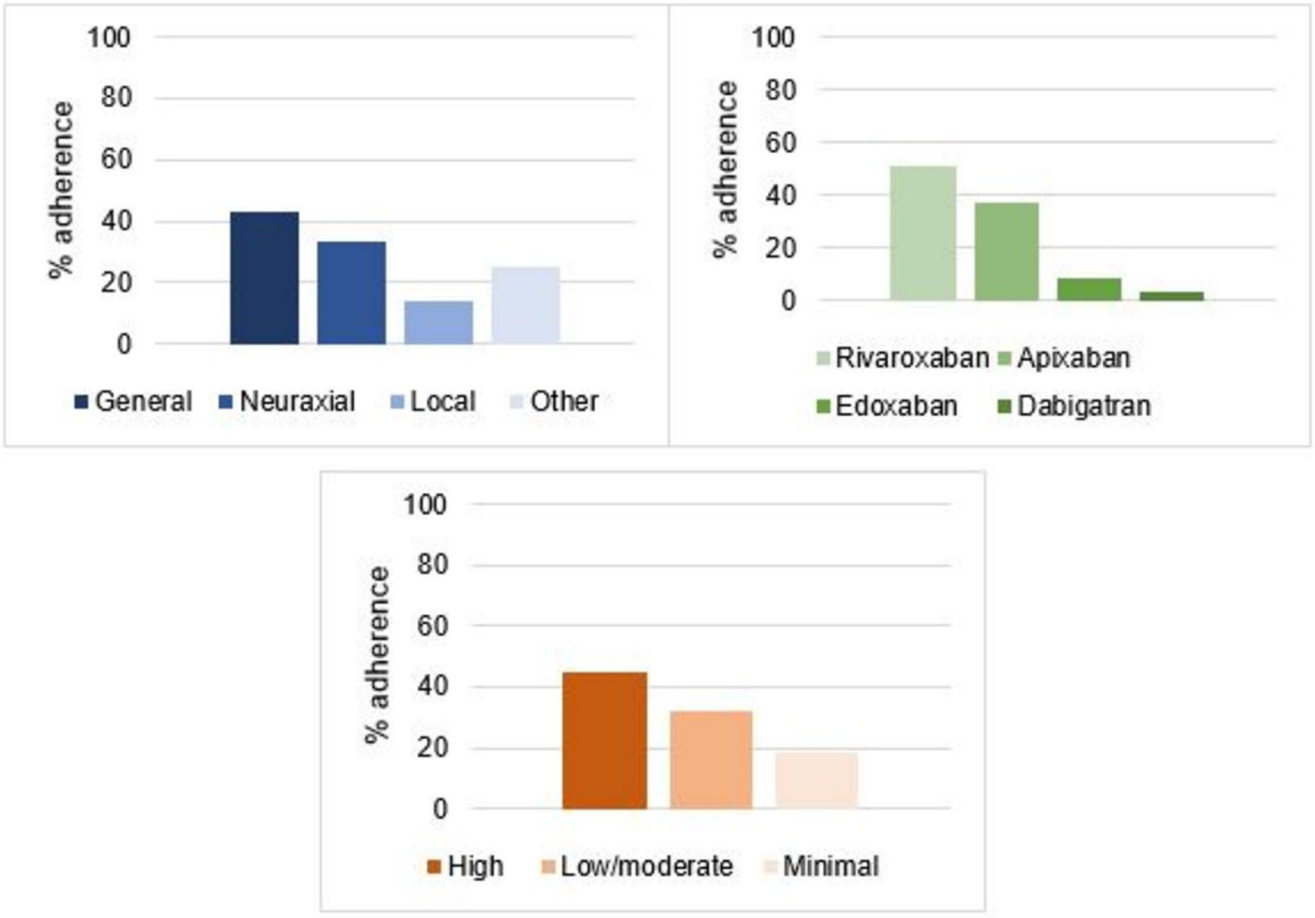
Proportions of patients whose DOAC management aligned with the ACCP guidelines according to the type of anaesthesia, type of DOAC and bleeding risk

Univariate analyses showed potential associations between treatment adherence and BMI (95%CI 1.0–1.1; *p* = 0.07), type of DOAC (OR = 0.83, 95%CI 0.65–1.04; *p* = 0.11), bleeding risk (OR = 0.54, 95%CI 0.40–0.72; *p* < 0.001) and type of anaesthesia (OR = 0.50, 95%CI 0.37–0.68; *p* < 0.001) (Table 3 and Fig. 3). Multivariate analyses showed that local anesthesia was more frequently used in situations of clinicians' non-adherence to institutional recommendations (OR = 0.30, 95%CI 0.14–0.66; *p* < 0.01) and with apixaban-treated patients than with those treated using rivaroxaban (OR = 0.57, 95%CI 0.33–0.97; *p* = 0.04) (Table 3).

**Table 3:** Logistic regression analyses for clinicians’ adherence to ACCP guidelines

**Variable**	**Adherent group**	**Non-adherent group**	**OR (Univariate)**	**OR (multivariate)**
Age (mean;SD)	73.84 (1.04)	73.89 (0.82)	1.0 (95%CI 0.98–1.0; *p* = 0.97)	-
Sex (%)	Female 35.75	Female 37.93	0.91 (95%CI 0.57–1.45; *p* = 0.69)	-
	Male 64.25	Male 62.07		
CHA_2_DS_2_VASc (mean;SD)	3.80 (0.13)	3.90 (0.14)	0.97 (95%CI 0.86–1.10; *p* = 0.62)	-
Antiplatelet agent (%)	Yes (89.66), No (10.34)	Yes (86.88), No (13.12)	0.76 (95%CI 0.37–1.56; *p* = 0.46)	-
DOAC indication (AF *vs* VTE) (%)	AF (71.55)	AF (71.95)	1.02 (95%CI 0.62–1.56; *p* = 0.94)	-
	VTE (28.45)	VTE (30.22)		
BMI (mean;SD)	27.09 (0.46)	26.09 (0.32)	1.04 (95%CI 1.0–1.1; *p* = 0.07)	1.05 (95%CI 0.99–1.1; *p* = 0.09)
DOAC type (%)	Rivaroxaban (37.07)	Rivaroxaban (28.05)	-	-
	Edoxaban (8.62)	Edoxaban (9.50)	0.69 (95%CI 0.29–1.60; *p* = 0.39)	0.76 (95%CI 0.31–1.87; *p* = 0.55)
	Apixaban (50.86)	Apixaban (58.37)	0.65 (95%CI 0.40–1.08; *p* = 0.10)	0.57 (95%CI 0.33–0.97; *p* = 0.04)
	Dabigatran (3.45)	Dabigatran (4.07)	0.64 (95%CI 0.19–2.22; *p* = 0.48)	0.52 (95%CI 0.14–1.93; *p* = 0.33)
Bleeding risk (%)	High bleeding risk (59.48)	High bleeding risk (38.46)	-	-
	Low/moderate bleeding risk (26.72)	Low/moderate bleeding risk (29.41)	0.59 (95%CI 0.34–1.00; *p* = 0.05)	0.54 (95%CI 0.46–1.50; *p* = 0.67)
	Minimal bleeding risk (13.79)	Minimal bleeding risk (3.21)	0.27 (95%CI 0.15–0.52; *p* < 0.001)	0.53 (95%CI 0.24–1.13; *p* = 0.1)
Type of anaesthesia (%)	General (83.62)	General (58.82)	-	-
	Neuraxial (4.31)	Neuraxial (4.52)	0.67 (95%CI 0.22–2.02; *p* = 0.48)	0.56 (95%CI 0.18–1.76; *p* = 0.32)
	Local (11.21)	Local (35.29)	0.22 (95%CI 0.12–0.43; *p* < 0.001)	0.30 (95%CI 0.14–0.66; *p* < 0.01)
	Other (0.86)	Other (1.36)	0.45 (95%CI 0.05–4.36; *p* = 0.49)	0.59 (95%CI 0.05–6.75; *p* = 0.67)

*DOAC* direct oral anticoagulant*, BMI* body mass index, *AF* atrial fibrillation, *VTE* venous thromboembolism

Since recent CrCl levels were only available for 211 (62.6%) patients, we ran a separate statistical analysis. Univariate analyses did show a potential association between categories of renal failure and clinicians’ adherence to institutional recommendations (OR = 0.66, 95%CI 0.37–1.17; *p* = 0.16) (Supplementary Table 4, Additional file 1). However, multivariate analyses using renal clearance did not reveal any significant associations with adherence (OR = 0.80, 95%CI 0.43–1.5; *p* = 0.49). This subgroup analysis showed the same results as the main multivariate analysis, except that BMI was significantly associated with a slight increase in clinicians’ adherence (Supplementary Table 4, Additional file 1).

### Postponements of surgical procedures

Overall, 45 procedures were postponed (13.3% of total procedures) (Table 4). We identified three different causes of deferrals: i) related to anticoagulation mismanagement according to the institutional recommendations (*n* = 11); ii) related to another known cause (Supplementary Table 5, Additional file 1*n* = 15); and iii) related to an unknown cause (*n* = 19). Among postponements directly related to clinicians’ non-adherence to recommendations, three postponements were related to patients’ behaviours (misunderstanding of instructions).

**Table 4:** Causes of procedure postponement

**Cause of postponement**	***n* = 45**
Known cause unrelated to anticoagulation management (n, %)	15 (33.3)
Unknown cause (not enough information in patient files) (n, %)	19 (42.2)
**Known cause related to suboptimal anticoagulation management (n, %)**	11 (24.4)
Late interruption (n, %)	4 (36.4)
No interruption (n, %)	3 (27.3)
Unrecommended heparin bridging (n, %)	4 (36.4)

Non-recommended heparin bridging was the major cause of postponements to surgery related to anticoagulation mismanagement (with anti-Xa measured and values deemed unsuitable for surgical procedures; Supplementary Table 6, Additional file 1), followed by the failure to interrupt DOACs before the procedure and a failure to interrupt DOACs early enough (Table 4).

## Discussion

The present study’s main objective was to evaluate clinicians’ adherence to our institution’s recommendations for the preoperative management of DOACs, which are largely based on the 2022 ACCP guidelines [5]. This evaluation took place over a two-year period in our tertiary hospital centre. Surprisingly, only one-third of the patients were treated in adherence with the DOAC management protocol suggested to clinicians in our institutional recommendations. Although patient recruitment occurred in 2019 and 2020, our recommendations were very similar to the 2022 ACCP guidelines, allowing a valid evaluation with them. It is of note that our institutional recommendations suggest heparin bridging for patients who have recently undergone a thromboembolic event (< 3 months previously), whereas the ACCP guidelines do not recommend this [5]. However, non-adherence to heparin bridging recommendations involved less than 1% of the 65.1% of non-adherent cases. Other exceptions were the recommendations for minimal-bleeding-risk procedures, which were included in the low-risk procedures category, as in the PAUSE study [7]. Patients were therefore considered to be non-adherent when clinicians did not stop their DOAC treatment for minimal-bleeding-risk procedures (except flutter ablations and cardioversions, which were excluded from the study).

In the recent ACCP guidelines, DOACs can be continued in cases of minimal bleeding risk or according to the clinician’s judgment [5]. However, only about 6% of the patients had not had their anticoagulant interrupted when a continuation was not recommended, suggesting a minimal influence on the final result.

These results contrasted with the 94% adherence observed in the PAUSE study [7]. However, our study reflected a real-life setting that cannot be compared with the adherence to a study protocol. Premature interruption and heparin bridging (when not recommended) were responsible for more than half of the observed management failures by clinicians. Fear of bleeding or thromboembolic events could have driven this approach by clinicians despite data from the PAUSE study showing low rates of thromboembolic and haemorrhagic complications associated with this preoperative management of DOACs [7]. It is possible that clinicians still lack knowledge about our institutional recommendations, although they have been widely distributed as a pocket guide since 2015. Our findings could reflect a lack of confidence in these guidelines as some clinicians argue that the PAUSE study did not include enough patients with a high bleeding risk. Indeed, only a third of the patients it included underwent high-bleeding-risk surgery, with most being cardiac surgery [5]. Clinicians could thus be less prone to following the ACCP guidelines when dealing with high-bleeding-risk surgeries; therefore, they increased the delay between the last DOAC dose and the surgery and/or the prescription of heparin bridging. However, the fact that our study’s adherence rates were low for all bleeding risk categories argues against this. Local anaesthesia was associated with a 70% reduction in adherence to our institution’s recommendations. Although the 2022 ACCP guidelines do not suggest specific strategies for local anaesthesia, this lack of adherence is not surprising because the European Society of Anaesthesia (ESA) guidelines for regional anaesthesia do not propose discontinuation of anticoagulants for superficial nerve blocks, only for deep nerve blocks [10]. Thus, some clinicians probably decided to maintain DOAC therapy during the intervention, as the ESA guidelines recommend. Interestingly, apixaban treatment was also associated with less adherence than rivaroxaban treatment, even after multivariate analysis. Therefore, patient morbidity or procedure risk in patients under apixaban cannot be claimed as causes of poor adherence. Although a complete explanation of this observation remains elusive, we can hypothesise that the twice-daily administration of apixaban may have appeared more confusing for clinicians attempting to manage the preoperative interruption of the drug.

The percentage of postponed procedures (13.3% of all procedures) was slightly higher than the rate found in high-income countries (< 10%) [11]. The preoperative mismanagement of DOACs was directly responsible for the postponement of 11 procedures (25% of all postponed procedures). However, three postponements were not directly related to clinicians’ decisions but rather to patients misunderstanding their recommendations. Although this rate seems low, it underlines how important it is for clinicians to adequately transmit information to patients. For the remaining cases, our results suggested that clinicians’ better adherence to guidelines could substantially decrease rates of postponed procedures.

The present study had some limitations. Its retrospective design revealed that DOAC plasma levels had not been systematically measured, and we do not know whether these could have influenced clinicians’ periprocedural management. The 2022 ACCP guidelines do not recommend routinely measuring DOAC plasma levels before surgery [5], even though some authors think this should be the case [12]. Increasing evidence shows associations between DOAC plasma levels and clinical outcomes, particularly bleeding [13, 14], and those levels could play a role in specific populations with comorbidities that can modify DOAC elimination. Our retrospective design did not allow us to reliably identify all the determinants of clinicians’ non-adherence to guidelines, specifically whether clinicians failed to follow the recommendations because they did not know about them or whether they voluntarily chose to ignore them. Generalising our results would be questionable as the preoperative management of DOACs very often relies on a clinician’s judgement and habits despite institutional recommendations. Indeed, it would have been interesting to compare our results against those obtained in other hospital centres, and to follow up and compare adherent *vs* non-adherent clinicians in terms of their patients’ post-surgical clinical outcomesIn addition, we did not test the clinicians’ familiarity, trust and knowledge of guidelines that were recently established which may have influenced adherent rates. Last but not least, the data regarding the surgical sites, which can impact the clinician’s adherence (e.g. neurosurgery), were not available for the analysis.

## Conclusion

This study of 337 consecutive patients treated with DOACs and who underwent elective surgery found that the preoperative anticoagulation management of approximately two-thirds of them failed to comply with our in-house institutional recommendations. To the best of our knowledge, this was the first clinical study addressing the issue of clinicians’ adherence to guidelines for the preoperative management of DOACs. Going beyond the issue of whether clinicians are knowledgeable about guidelines or have them available, this study questions how generalisable guidelines are in a tertiary hospital managing many highly polymorbid patients.

### Supplementary Information

**Additional file 1: Supplementary Table 1.** Perioperative anticoagulation management according to the American College of Chest Physicians Clinical Practice Guidelines. Adapted from [1]. DOAC = direct oral anticoagulant. CrCl = creatinine clearance. O = on DOAC. X = not on DOAC. **Supplementary Table 2.** Risk Stratification for Procedural Bleed Risk as suggested by the International Society on Thrombosis and Haemostasis (ISTH) Guidance Statement [2]. Adapted from [3]. **Supplementary Table 3.** Preoperative management for direct oral anticoagulants according to current international and local guidelines. GFR: glomerular filtration rate according to Cokroft-Gault. **Supplementary Table 4.** Multivariate model of clinicians’ adherence to ACCP guidelines for those with available creatinine clearance. **Supplementary Table 5.** Cause of postponement unrelated to anticoagulation management. **Supplementary Table 6.** Anti-Xa measurements leading to procedure postponement.

## Data Availability

The datasets used and/or analysed during the current study are available from the corresponding author on reasonable request.

## Abbreviations

ACCP: American College of Chest Physicians
AF: Non-valvular atrial fibrillation
CrCl: Creatinine clearance
DOACs: Direct oral anticoagulants
DVT: Deep vein thrombosis
ESA: European Society of Anaesthesia
ISTH: International Society on Thrombosis and Haemostasis
PE: Pulmonary embolism
VKA: Vitamin K antagonists

## References

[1] January CT, Samuel WL, Hugh C, Chen LY, Cigarroa JE, Cleveland JC (2019). AHA/ACC/HRS Focused Update of the 2014 AHA/ACC/HRS guideline for the management of patients with atrial fibrillation: a report of the American College of Cardiology/American Heart Association task force on clinical practice guidelines and the heart rhythm society in collaboration with the society of thoracic surgeons. Circulation.

[2] Chen A, Stecker EA, Warden B (2020). Direct oral anticoagulant use: a practical guide to common clinical challenges. J Am Heart Assoc.

[3] Fowler AJ, Abbott TEF, Prowle J, Pearse RM (2019). Age of patients undergoing surgery. Br J Surg.

[4] Zulkifly H, Lip GYH, Lane DA (2018). Epidemiology of atrial fibrillation. Int J Clin Pract.

[5] Douketis JD, Spyropoulos AC, Murad MH, Arcelus JI, Dager WE, Dunn AS (2022). Executive summary: perioperative management of antithrombotic therapy: an American College of Chest physicians clinical practice guideline. Chest.

[6] Steffel J, Collins R, Antz M, Cornu P, Desteghe L, Haeusler KG (2021). 2021 European heart rhythm association practical guide on the use of non-vitamin K antagonist oral anticoagulants in patients with atrial fibrillation. Europace.

[7] Douketis JD, Spyropoulos AC, Duncan J, Carrier M, Le Gal G, Tafur AJ (2019). Perioperative management of patients with atrial fibrillation receiving a direct oral anticoagulant. JAMA Intern Med.

[8] Spyropoulos AC, Brohi K, Caprini J, Samama CM, Siegal D, Tafur A (2019). Scientific and standardization committee communication: guidance document on the periprocedural management of patients on chronic oral anticoagulant therapy: recommendations for standardized reporting of procedural/surgical bleed risk and patient-specific thromboembolic risk. J Thromb Haemost.

[9] https://www.hug.ch/sites/interhug/files/structures/angiologie_et_hemostase/documents/a65_acods2019_4.pdf n.d.

[10] Kietaibl S, Ferrandis R, Godier A, Llau J, Lobo C, Macfarlane AJ (2022). Regional anaesthesia in patients on antithrombotic drugs: Joint ESAIC/ESRA guidelines. Eur J Anaesthesiol.

[11] Kaddoum R, Fadlallah R, Hitti E, El-Jardali F, El Eid G (2016). Causes of cancellations on the day of surgery at a Tertiary Teaching Hospital. BMC Health Serv Res.

[12] Stretton B, Kovoor JG, Gupta AK, Ovenden C, Litwin P, Maddern GJ, et al. Making up for lost time: perioperative direct oral anticoagulant assay measurements. Preprints. 2022. 10.22541/au.166723035.55498329/v1.

[13] Eikelboom JW, Quinlan DJ, Hirsh J, Connolly SJ, Weitz JI (2017). Laboratory monitoring of non-vitamin K antagonist oral anticoagulant use in patients with atrial fibrillation: a review. JAMA Cardiol.

[14] Moner-Banet T, Alberio L, Bart P-A (2020). Does one dose really fit all? On the monitoring of direct oral anticoagulants: a review of the literature. Hamostaseologie.

