# Supplementary material for: Clinicians’ adherence to guidelines for the preoperative management of direct oral anticoagulants in a tertiary hospital: a retrospective study
Source: BMC Anesthesiol. 2023 Sep 15;23:314. doi: 10.1186/s12871-023-02276-w (PMC10503177; doi:10.1186/s12871-023-02276-w)
Supplement: Supplementary file 1 — Additional file 1: Supplementary Table 1. Perioperative anticoagulation management according to the American College of Chest Physicians Clinical Practice Guidelines. Adapted from [1]. DOAC = direct oral anticoagulant. CrCl = creatinine clearance. O = on DOAC. X = not on DOAC. Supplementary Table 2. Risk Stratification for Procedural Bleed Risk as suggested by the International Society on Thrombosis and Haemostasis (ISTH) Guidance Statement [2]. Adapted from [3]. Supplementary Table 3. Preoperative management for direct oral anticoagulants according to current international and local guidelines. GFR: glomerular filtration rate according to Cokroft-Gault. Supplementary Table 4. Multivariate model of clinicians’ adherence to ACCP guidelines for those with available creatinine clearance. Supplementary Table 5. Cause of postponement unrelated to anticoagulation management. Supplementary Table 6. Anti-Xa measurements leading to procedure postponement. [file 12871_2023_2276_MOESM1_ESM.docx]

| DOAC | Surgical procedure-associated bleeding risk | Preoperative DOAC interruption schedule | | | | | Day of surgical procedure (No DOAC) | Postoperative DOAC resumption schedule | | | |
| --- | --- | --- | --- | --- | --- | --- | --- | --- | --- | --- | --- |
|  |  | Day -5 | Day -4 | Day -3 | Day -2 | Day -1 |  | Day +1 | Day +2 | Day +3 | Day +4 |
| Apixaban | High | O | O | O | X | X |  | X | X | O | O |
|  | Low | O | O | O | O | X |  | O | O | O | O |
| Dabigatran (CrCl ≥ 50 ml/min) | High | O | O | O | X | X |  | X | X | O | O |
|  | Low | O | O | O | O | X |  | O | O | O | O |
| Dabigatran (CrCl < 50 ml/min) | High | O | O | O | X | X |  | X | X | O | O |
|  | Low | O | O | O | X | X |  | O | O | O | O |
| Rivaroxaban | High | O | O | O | X | X |  | X | X | O | O |
|  | Low | O | O | O | O | X |  | O | O | O | O |

**Supplementary Table 1.** Perioperative anticoagulation management according to the American College of Chest Physicians Clinical Practice Guidelines. Adapted from [1]. DOAC = direct oral anticoagulant. CrCl = creatinine clearance. O = on DOAC. X = not on DOAC.

**Supplementary table 2.** Risk Stratification for Procedural Bleed Risk as suggested by the International Society on Thrombosis and Haemostasis (ISTH) Guidance Statement [2]. Adapted from [3].

| High-bleed-risk surgery/procedure (30-day risk of  major bleeding ≥ 2%) | Major surgery with extensive tissue injury  Cancer surgery, especially solid tumour resection (lung, oesophagus, gastric, colon, hepatobiliary, pancreatic)  Major orthopaedic surgery, including shoulder replacement surgery  Reconstructive plastic surgery  Major thoracic surgery  Urological or GI surgery, especially anastomosis surgery  Transurethral prostate resection, bladder resection, or tumour ablation  Nephrectomy, kidney biopsy  Colonic polyp resection  Bowel resection  Percutaneous endoscopic gastrostomy placement, endoscopic  retrograde cholangiopancreatography  Surgery in highly vascular organs (kidneys, liver, spleen)  Cardiac, intracranial, or spinal surgery  Any major operation (procedure duration > 45 min)  Neuraxial anaesthesia  Epidural injections |
| --- | --- |
| Low-to-moderate-bleed-risk surgery/procedure  (30-day risk of major bleeding 0–2%) | Arthroscopy  Cutaneous/lymph node biopsies  Foot/hand surgery  Coronary angiography  GI endoscopy biopsy  Colonoscopy biopsy  Abdominal hysterectomy  Laparoscopic cholecystectomy  Abdominal hernia repair  Hemorrhoidal surgery  Bronchoscopy biopsy |
| Minimal-bleed-risk surgery/procedure (30-d  risk of major bleed approximately 0%) | Minor dermatological procedures (excision of basal and squamous cell, skin cancers, actinic keratoses, and premalignant or cancerous skin nevi)  Ophthalmologic (cataract) procedures  Minor dental procedures (dental extractions, restorations, prosthetics, endodontics), dental cleanings, fillings  Pacemaker or cardioverter-defibrillator device implantation |

**Supplementary Table 3.** Preoperative management for direct oral anticoagulants according to current international and local guidelines. GFR: glomerular filtration rate according to Cokroft-Gault.

| Guidelines  ​ | DOAC plasma monitoring  ​ | DOAC preoperative management |
| --- | --- | --- |
| Local guidelines (Geneva University Hospital)[4] | Only suggested for neuraxial anaesthesia and lumbar puncture otherwise not recommended | According to PAUSE protocole[5]  + heparin-briging in case of a recent thrombolic event < 3 months |
| 2022​ American College of chest physician (ACCP)[1] | Not recommended ​ | According to PAUSE protocole[5] |
| 2021 European Heart Rhythm Association​ (EHRA) [6] | Only if very high risk of accumulation (advanced renal failure) | According to PAUSE protocole[5] |
| ​  ​  2017 Groupe d'Intérêt en Hémostase Périopératoire (GIHP) ​[7]  ​ | Not indicated unless accumulation or prolonged elimination is suspected. Do not perform spinal anaesthesia or deep block techniques in patients for whom detectable DOAC blood concentration is possible (insufficient time to stop)​. | Low bleeding risk :  1) apixaban, rivaroxaban, edoxaban, dabigatran) : not taken the night before or the morning of the invasive procedure  High bleeding risk :  1) apixaban, rivaroxaban, edoxaban and GFR>30ml/min : last take at d-3  2) dabigatran and GFR 30-49 ml/min last take at d-5  3) dabigatran and GFR>50 ml/min last take at d-4 |

**Supplementary Table 4.** Multivariate model of clinicians’ adherence to ACCP guidelines for those with available creatinine clearance.

| **Variable** | **Categories** | **OR (Univariate)** | **OR (multivariate)** |
| --- | --- | --- | --- |
| Renal category | CrCl ≥ 50 ml/min |  |  |
|  | CrCl = 30–49 ml/min | 0.47 (95%CI 0.21–1.01; *p* = 0.05) | 0.66 (95%CI 0.28–1.55; *p* = 0.34) |
|  | Cl < 30ml/min | 0.92 (95%CI 0.21–4.0; *p* = 0.92) | 0.96 (95%CI 0.18–5.01; *p* = 0.96) |
| Age | - | 0.99 (95%CI 0.96–1.02; *p* = 0.53) | - |
| Sex | - | 1.02 (95%CI 0.56–1.85; *p* = 0.95) | - |
| CHA_2_DS_2_VASc | - | 0.96 (95%CI 0.83–1.12; *p* = 0.64) | - |
| Antiplatelet agent | - | 0.52 (95%CI 0.21–1.28; *p* = 0.16) | 0.46 (95%CI 0.17–1.20; *p* = 0.12) |
| BMI | - | 1.1 (95%CI 1.03–1.17; *p* < 0.01) | 1.08 (95%CI 1.01–1.15; *p* < 0.05) |
| ACOD type | Rivaroxaban | - | - |
|  | Edoxaban | 0.67 (95%CI 0.24–1.86; *p* = 0.44) | 0.67 (95%CI 0.22–2.04; *p* = 0.49) |
|  | Apixaban | 0.63 (95%CI 0.34–1.17; *p* = 0.15) | 0.45 (95%CI 0.22–0.9; *p* < 0.05) |
|  | Dabigatran | 0.76 (95%CI 0.20–2.84; *p* = 0.69) | 0.44 (95%CI 0.10–1.87; *p* = 0.26) |
| Bleeding risk | High bleeding risk | - | - |
|  | Low/moderate bleeding risk | 0.72 (95%CI 0.38–1.37; *p* = 0.32) | 1.31 (95%CI 0.60–2.85; *p* = 0.49) |
|  | Minimal bleeding risk | 0.29 (95%CI 0.11–0.76; *p* < 0.05) | 0.55 (95%CI 0.17–1.78; *p* = 0.32) |
| Type of anaesthesia | General | - |  |
|  | Neuraxial | 0.66 (95%CI 0.19–2.28; *p* = 0.51) | 0.66 (95%CI 0.18–2.46; *p* = 0.54) |
|  | Local | 0.17 (95%CI 0.06–0.46; *p* < 0.001) | 0.21 (95%CI 0.07–0.67; *p* < 0.05) |
|  | Other | 0.66 (95%CI 0.05–7.42; *p* = 0.74) | 0.97 (95%CI 0.06–15.34; *p* = 0.98) |

**Supplementary Table 5.** Cause of postponement unrelated to anticoagulation management.

| Cause of postponement unrelated to anticoagulation management | n = 15 |
| --- | --- |
| Surgery finally not indicated | 2 |
| COVID | 2 |
| Anaesthesia assessment requested for sudden massive weight gain | 1 |
| Acute heart failure | 1 |
| Surgeon deemed anticoagulation poorly managed | 1 |
| Transient ischemic attack | 1 |
| Infection | 4 |
| Issue with patient’s pacemaker | 1 |
| Sub-ileus | 1 |
| Lower gastrointestinal bleeding | 1 |

**Supplementary Table 6.** Anti-Xa measurements leading to procedure postponement.

|  |  |
| --- | --- |
| Compound | **Anti-Xa** |
| Fondaparinux | 0.22 IU/ml |
| Unfractionated heparin | 0.34 IU/ml |
| Unfractionated heparin | 0.18 IU/ml |
| Nadroparin | 0.23 IU/ml |

**References**

1. Douketis JD, Spyropoulos AC, Murad MH, Arcelus JI, Dager WE, Dunn AS, et al. Executive Summary: Perioperative Management of Antithrombotic Therapy: An American College of Chest Physicians Clinical Practice Guideline. Chest. 2022;162:1127–39.

2. Spyropoulos AC, Brohi K, Caprini J, Samama CM, Siegal D, Tafur A, et al. Scientific and Standardization Committee Communication: Guidance document on the periprocedural management of patients on chronic oral anticoagulant therapy: Recommendations for standardized reporting of procedural/surgical bleed risk and patient-specific thromboembolic risk. Journal of Thrombosis and Haemostasis. 2019;17:1966–72.

3. Barnes GD, Mouland E. Peri-Procedural Management of Oral Anticoagulants in the DOAC Era. Prog Cardiovasc Dis. 2018;60:600–6.

4. https://www.hug.ch/sites/interhug/files/structures/angiologie_et_hemostase/documents/a65_acods2019_4.pdf.

5. Douketis JD, Spyropoulos AC, Duncan J, Carrier M, Le Gal G, Tafur AJ, et al. Perioperative Management of Patients With Atrial Fibrillation Receiving a Direct Oral Anticoagulant. JAMA Intern Med. 2019;179:1469–78.

6. Steffel J, Collins R, Antz M, Cornu P, Desteghe L, Haeusler KG, et al. 2021 European Heart Rhythm Association Practical Guide on the Use of Non-Vitamin K Antagonist Oral Anticoagulants in Patients with Atrial Fibrillation. Europace. 2021;23:1612–76.

7. Albaladejo P, Bonhomme F, Blais N, Collet J-P, Faraoni D, Fontana P, et al. Management of direct oral anticoagulants in patients undergoing elective surgeries and invasive procedures: Updated guidelines from the French Working Group on Perioperative Hemostasis (GIHP) - September 2015. Anaesth Crit Care Pain Med. 2017;36:73–6.
